# Patient Perspectives on Sharing Anonymized Personal Health Data Using a Digital System for Dynamic Consent and Research Feedback: A Qualitative Study

**DOI:** 10.2196/jmir.5011

**Published:** 2016-04-15

**Authors:** Karen Spencer, Caroline Sanders, Edgar A Whitley, David Lund, Jane Kaye, William Gregory Dixon

**Affiliations:** ^1^ Arthritis Research UK Centre for Epidemiology Manchester Academic Health Sciences Centre The University of Manchester Manchester United Kingdom; ^2^ National Institute for Health Research (NIHR) School for Primary Care Research Manchester Academic Health Sciences Centre The University of Manchester Manchester United Kingdom; ^3^ Department of Management London School of Economics and Political Science London United Kingdom; ^4^ HW Communications Ltd Lancaster United Kingdom; ^5^ Centre for Health, Law and Emerging Technologies Nuffield Department of Population Health University of Oxford Oxford United Kingdom; ^6^ Health eResearch Centre, Farr Institute Manchester Academic Health Sciences Centre The University of Manchester Manchester United Kingdom; ^7^ Department of Rheumatology Salford Royal National Health Service (NHS) Foundation Trust Salford United Kingdom

**Keywords:** eHealth, data sharing, public trust, consent

## Abstract

**Background:**

Electronic health records are widely acknowledged to provide an important opportunity to anonymize patient-level health care data and collate across populations to support research. Nonetheless, in the wake of public and policy concerns about security and inappropriate use of data, conventional approaches toward data governance may no longer be sufficient to respect and protect individual privacy. One proposed solution to improve transparency and public trust is known as Dynamic Consent, which uses information technology to facilitate a more explicit and accessible opportunity to opt out. In this case, patients can tailor preferences about whom they share their data with and can change their preferences reliably at any time. Furthermore, electronic systems provide opportunities for informing patients about data recipients and the results of research to which their data have contributed.

**Objective:**

To explore patient perspectives on the use of anonymized health care data for research purposes. To evaluate patient perceptions of a Dynamic Consent model and electronic system to enable and implement ongoing communication and collaboration between patients and researchers.

**Methods:**

A total of 26 qualitative interviews and three focus groups were conducted that included a video presentation explaining the reuse of anonymized electronic patient records for research. Slides and tablet devices were used to introduce the Dynamic Consent system for discussion. A total of 35 patients with chronic rheumatic disease with varying levels of illness and social deprivation were recruited from a rheumatology outpatient clinic; 5 participants were recruited from a patient and public involvement health research network.

**Results:**

Patients were supportive of sharing their anonymized electronic patient record for research, but noted a lack of transparency and awareness around the use of data, making it difficult to secure public trust. While there were general concerns about detrimental consequences of data falling into the wrong hands, such as insurance companies, 39 out of 40 (98%) participants generally considered that the altruistic benefits of sharing health care data outweighed the risks. Views were mostly positive about the use of an electronic interface to enable greater control over consent choices, although some patients were happy to share their data without further engagement. Participants were particularly enthusiastic about the system as a means of enabling feedback regarding data recipients and associated research results, noting that this would improve trust and public engagement in research. This underlines the importance of patient and public involvement and engagement throughout the research process, including the reuse of anonymized health care data for research. More than half of patients found the touch screen interface easy to use, although a significant minority, especially those with limited access to technology, expressed some trepidation and felt they may need support to use the system.

**Conclusions:**

Patients from a range of socioeconomic backgrounds viewed a digital system for Dynamic Consent positively, in particular, feedback about data recipients and research results. Implementation of a digital Dynamic Consent system would require careful interface design and would need to be located within a robust data infrastructure; it has the potential to improve trust and engagement in electronic medical record research.

## Introduction

The National Health Service (NHS) provides health care for over 60 million citizens throughout their lives, with vast amounts of information about patients’ treatments and outcomes collected in their medical records. Such real-world data is an important asset for UK health research: patients’ "cradle to grave" records are increasingly captured within electronic patient record (EPR) systems, providing the opportunity to anonymize patient-level health care data and collate across populations to support research. The importance and vast opportunity of sharing health care data for research is explicit within the UK government’s Strategy for UK Life Sciences [1]. This has been supported by the recent cross-funder investment to establish the Farr Institute, a network of academic eHealth Centres of Excellence [2].

There is a reasonable expectation in society that the sensitive and personal nature of health care data requires that it should be carefully managed and access to it should be restricted only to those with a legitimate purpose. As a consequence, the UK legal regime has conditions for the use of health care data but at the same time allows certain exemptions for research carried out in the public interest. Under the Data Protection Act (1998), patient consent is not required when anonymized data are used for research, although there may be societal concerns that “go beyond compliance with the requirements of formal regulation” [3]. Health care data are highly personal and are usually of a sensitive nature, making it difficult to anonymize data effectively to maintain the privacy of patients. The Data Protection Act also has a fair processing obligation that requires patients to be informed about what happens to their data that applies to all kinds of data [4]. This aligns with a shared societal expectation that patients have a right to know how their data are being used and should be given the opportunity to consent but also object to their data being shared with others, even in the case of “anonymized” data.

The UK government’s care.data initiative, a program intended to enable sharing of anonymized primary care health records with "researchers and organizations outside the NHS" [5] for research and service improvement, was paused in 2014 due to a loss of public trust [3]. Trust is often taken as the measure of an individual’s willingness to be vulnerable to the actions of another person on the basis that the trustee will act according to the trustor’s confident expectations [6]. Different forms of trust include deterrence-based trust, where the trustor is confident that the trustee will act as expected because sanctions for breach of trust are very high; calculus-based trust, where the trustor "evaluates" the reputation/certification of the trustee; relational trust that arises when repeated interactions have gone well; and institution-based trust, which combines calculus and relational trust through the proxy of the trusted institution [7]. Although health care institutions can normally assume a high level of institutional trust [8], if lost, it can be difficult to repair [9]. Reasons cited for this loss of public trust included concerns that personal health care data might be used inappropriately (eg, sharing with insurance companies or being sold for profit [10], as well as lack of clarity as to how patients should opt out). The population-level approach of the above campaign failed to reassure many patients about potential misuse of data and, although recent studies have shown that most patients support confidential reuse of health data, concerns have also arisen surrounding security, privacy, and control over access of EPRs [11-13]. Previous research has highlighted that the UK public has little knowledge of how their EPRs are used for medical research purposes [13] and the lack of transparency and engagement with patients is viewed to undermine public trust with implications for acceptable models of consent [14-16]. The Nuffield Council on Bioethics, in their review of the care.data plans, recommended that health authorities track the use of patient data, give people greater access, and say how their data is used [17]. This is important for maintaining trust and requires the opportunity and process for opting out of data sharing to be clear. Furthermore, an independent review of the care.data program by the National Data Guardian has asked of any future system, "How can patients check, update, or change their preferences and see that their choices have been respected?" [18].

One proposed solution for the problems outlined above is known as Dynamic Consent, which uses information technology (IT) to facilitate a more explicit and accessible opportunity to opt out, whereby patients can tailor preferences about whom they share their data with, and can change their preferences reliably at any time preventing any further data sharing [19,20]. This is achieved technically by binding patient information with consent expressions [19]. In addition, via the same digital interface, patients can be provided with information as to the recipients of their data plus other information, such as results of research derived from their data contribution. Demonstrating to patients how sharing their data has contributed to improved care within the population could build community trust, and show how patients are already contributing to research within the NHS: a pledge within the NHS Constitution [21]. A prototype Dynamic Consent interface has been developed by the Ensuring Consent and Revocation (EnCoRe) project [20]. This was initially designed in the context of biobanking to allow patients to consent for the collection of biobank tissue and data, but the same principles, architecture, and philosophy could be used to facilitate the trusted sharing of EPR data for research. Implementing such a system for this purpose, however, faces some unknowns. While previous surveys have suggested that patients are willing to share their EPR for research [11], would they wish to express consent preferences using a digital system? Would patients value feedback information about who the recipients are and the results of the research via such a system? There is also a need to address the feasibility and barriers for using such a system, and how it could best be implemented.

The purpose of this study was to undertake qualitative research to (1) explore patient perspectives on the use of anonymized health data for research purposes and (2) to evaluate patient perceptions of a Dynamic Consent model and electronic system to enable and implement ongoing communication and collaboration between patients and researchers in this context.

## Methods

### Participants and Methods

A combination of qualitative in-depth interviews and focus groups were used to first explore patients’ perspectives on the use of anonymized personal health data for research, before introducing the model of Dynamic Consent and feedback and seeking patients’ views. Interviews and focus groups were also conducted to seek patient views on an electronic prototype system to collect consent and provide feedback. Focus groups are considered a valuable approach for exploring a range of public and patient views in health research, especially where the goal is to explore and develop a new intervention or service [22]. Combining interviews and focus groups enabled us to maximize recruitment because people could choose whether they wanted to take part in an interview or focus group. Interviews were effective in allowing in-depth discussions related to personal views and experiences. The interaction within focus groups generated some level of debate and consensus, as well as creative ideas about data and information sharing and the potential use of an electronic system.

Participants were recruited from a rheumatology outpatient clinic in a large teaching hospital (n=35) and from a patient and public involvement (PPI) health research network (n=5), both based in Salford, Greater Manchester in the United Kingdom. Within the clinic, unselected patients were identified by members of the clinical team and directed to the research associate (KS) for further information. All participants were provided with a patient information sheet describing the study. The sample was to some extent a convenience sample based on who responded to advertisements via the PPI network. However, we were able to purposively sample via the outpatient clinic to ensure maximum variation, including a mixture of men and women of various ages with varied levels of illness and health care experiences [23]. The final sample also had varied occupational, educational, and social circumstances, which were referred to in interviews and focus group discussions.

Three focus groups were conducted, consisting of 4-6 participants along with a moderator and note taker, and lasted approximately 90 minutes. Participants were organized into focus groups pragmatically according to when they consented and were available. A total of 26 semistructured interviews were conducted with patients, each lasting between 45 and 60 minutes. The focus groups and interviews were audiotaped with permission from participants; written informed consent was obtained prior to the start of any discussion. The study received ethical approval from Liverpool East Research Ethics Committee (Ref: 13/NW/0722). A patient and public involvement group comprised of five members was established at the start of the project. This group convened quarterly to inform aspects of the study, such as the design of information and interview guides, and to discuss and refine emerging findings from the focus groups and interviews.

### Procedure for Interviews/Focus Groups

**Figure 1:**
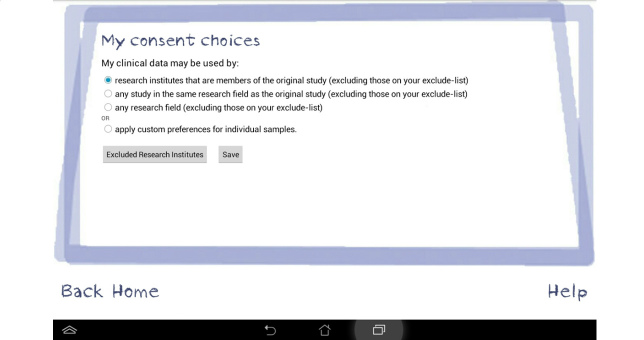
Screenshot of the Dynamic Consent prototype interface: My consent choices.

**Figure 2:**
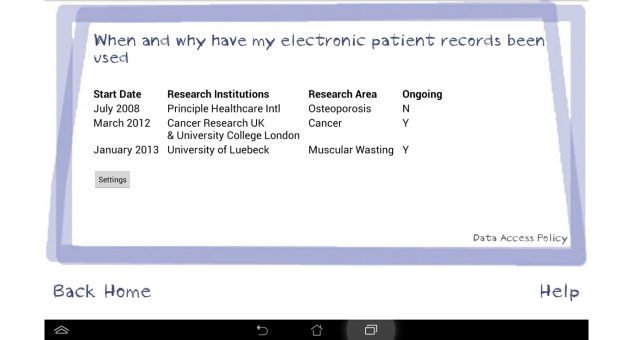
Screenshot of the Dynamic Consent prototype interface: When and why have my electronic patient records been used.

**Figure 3:**
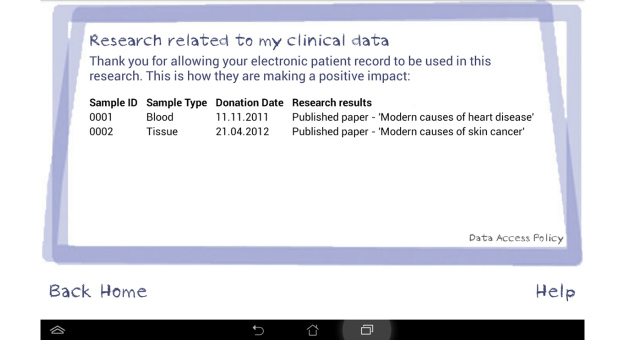
Screenshot of the Dynamic Consent prototype interface: Research related to my clinical data.

An interview/focus group topic guide was developed initially from the literature and subsequent topics were added if they arose during data collection. Topics discussed during the interviews and focus groups included the following: previous knowledge and understanding of how health data are stored and shared beyond the NHS, views and concerns regarding the storage and sharing of EPRs for research purposes, willingness of participants to share their health data and with whom, views about a Dynamic Consent model for reuse of anonymized health data, and views about a prototype electronic system for Dynamic Consent using a tablet device. As we were unsure as to the level of knowledge that participants held relating to their electronic patient records and how they might be anonymized and collated to benefit research, we developed a short 5-minute film that informed participants of current practice within the United Kingdom [24]. Included were visual examples of a clinical consultation involving entry of patient-level data into an EPR system followed by large anonymized datasets being used by university researchers. This was presented on the tablet device during individual interviews and via a projector during focus group discussions. Following initial discussion focused on understanding and views about storage and use of health data, the moderator introduced the Dynamic Consent prototype on a tablet interface with touch screen technology. The interface screens included the ability for patients to state their willingness (or not) to share their anonymized records with specific groups, for example research institutes or private companies—entitled *My consent choices*. Additional screens provided details of which groups had accessed their shared data, research studies using participants’ data, as well as links to published research and relevant news items (see Figures 1-3 for screenshots of the Dynamic Consent interface). The prototype interface was intended to provide sufficient detail to elicit patients’ views about the concept of Dynamic Consent. Details within the interface, such as how best to categorize research groups or optimal methods for patient feedback, were not tested but will form the basis of future research.

### Data Analysis

Interviews and focus groups were transcribed verbatim and NVivo version 10 software (QSR International) was used to facilitate analysis. Data were analyzed thematically using some key techniques of a grounded theory approach, including open coding and constant comparison to identify key (ie, selective) codes [25]. An iterative and inductive approach to analysis was followed so that analysis started in parallel with the data collection; initial results informed subsequent data collection as themes and issues were identified and informed further questions and probing around these emerging themes. For example, a key focus for initial discussions had been the model of Dynamic Consent and the associated interface to specify preferences. However, initial findings demonstrated that while people valued the potential for an opportunity to opt out of specific research, they were more enthusiastic to discuss the research feedback components of the system. Further discussions and questions about this enabled understanding of why this component was considered a priority for a diverse group of participants. Memos and documents were written about emerging categories, to summarize a point, to critique information, and to relate emergent theories to existing literature [25]. Authors KS, CS, and WGD met on a regular basis to discuss the development of codes, themes, categories, and theories about the phenomenon being studied. Recruitment ceased once data saturation was established.

## Results

### Overview

Of the 40 participants, 23 (58%) were women and 17 (43%) were men. Ages ranged from 23 to 88 years (mean 61, SD 13). With the exception of one white Canadian participant, participants described themselves as white British. All were suffering from a chronic rheumatic disease. Three key themes characterized participants’ views on the use of anonymized EPRs for medical research and their perceptions of Dynamic Consent: (1) the role of trust and perceived social responsibility to share health data, (2) transparency through Dynamic Consent and patient feedback and the potential for enhanced control and patient engagement, and (3) operational and technological scope and challenges for participation.

### The Role of Trust and Perceived Social Responsibility to Share Health Data

Individuals often indicated a high degree of trust in the NHS, for example stating, "I trust the NHS to store my information confidentially" (Participant #4) or "I'm generally quite trustful of hospitals and [general practitioners] GPs" (Participant #11). These and additional comments (see Textbox 1) indicate a sense of institutional trust in the health care system, as well as medical professionals. Most participants felt confident that electronic health records were managed securely and anonymity was preserved when used for research. Respondents tended to express a greater concern about security of financial data compared to health data. There was acknowledgement that there may have been some decline in public trust of the NHS and the medical profession in the wider population, but a number of people viewed the media to be responsible for overinflating a sense of public concern due to the "negative press coverage" (Participant #2) allowing "distractions from actual issues" (Participant #5). While the majority expressed satisfaction toward governance arrangements within the NHS, there was an expressed view that no system could be completely secure. A small minority of participants described concern about risk to their privacy, speculating that patients with more sensitive health conditions may be "more guarded of what happens with their health information" (Participant #11) due to fear of stigmatization. There was a general fear of detrimental consequences if data were to fall into the wrong hands, such as insurance companies, suggesting a more nuanced calculative sense of trust that went beyond trust in health care institutions. However, 39 out of 40 (98%) participants considered that the benefits of storing and sharing EPRs for medical research outweighed any perceived risk in terms of data security. Most participants appreciated the importance of medical research and the importance of sharing their EPR for the benefit of medical progress and the health of future generations (see Textbox 1). Only one participant held a contrary view, preferring not to share his data:

I would hate for my health details to be in there [national database]...It would be a good idea, but that’s in a really nice ideal world, and it’s not an ideal world. I would opt out. It’s not that I don’t trust the NHS, it’s that I don’t trust, you know, people…people make mistakes.

Participant #15

The discussions did not raise issues around different levels of anonymization or the potential for reidentification of patient identity through unique patterns of clinical history.

**Quotations representing the role of trust and perceived social responsibility to share health data.**

If researchers or health care professionals, or anybody were to look at my own personal records...I trust that they [NHS] have those skills to keep it anonymized.

Participant #2

I think most of my electronic record is pretty safe in the NHS, I trust them to protect my identity and look after the information.

Participant #18

I would never do personal banking because I'm of the age group that doesn’t trust things. But things like National Health, I would say yes.

Participant #14

There’s always going to be the pros and cons with [storing and sharing health data]. However, for me personally, the pros outweigh the cons.

Participant #5

I don’t care what people know about my health...I suppose for insurance for stuff and things like that, could bother some people.

Participant #12

I mean I can trust the doctors and all...but other people, no. Once it leaves the NHS, I’d be wondering where it’s going and who’s looking at it.

Participant #19

Once you have been in receipt of the excellent kind of care and treatment that I've had, I think you have a social responsibility that if you can help the next generation by having your information provided to the researchers to [do] some good.

Focus group #3

I am happy to share my health records. As long as it benefits other people.

Participant #28

I understand that advances can’t be done in medical science, unless people like me and others are taking part in research.

Participant #1

### Transparency Through Dynamic Consent and Patient Feedback, and the Potential for Enhanced Control and Patient Engagement

Despite the high level of institutional trust and sense of social responsibility, participants reported low levels of awareness about how their personal health data was currently stored and shared for medical research. Importantly, respondents highlighted that fear can come from the unknown causing people to be "very fearful, because you don’t know what’s going on, you don’t know if it’s identifiable" (Focus group #1). Some respondents referred to the need for greater information so that "you’re dealing with the information, rather than all these things that might be not true" (Participant #29), again highlighting the limits in high-level and more abstract institutional trust. The desire for greater transparency and engagement regarding the use of their data was reflected in repeatedly positive responses regarding the potential use of the digital interface as a useful tool to enable insight into how data is used for specific research studies. Respondents were mostly positive about the potential use of the interface to enable greater control over consent for specific studies because "it gives you choices" (Focus group #1), although some were happy to share their data without wanting to engage further (see below). Despite a clear introduction, patients did not talk about the time-varying nature of consent preferences, instead talking about the value of using the system to make a one-off decision if they wanted to opt out. Most respondents did not raise concerns about changing their minds at a later point and this was not explicitly asked about by researchers. Where respondents did give an example of wanting to reverse inclusion of their data, they assumed this would be possible. Many respondents thought they would try out using the system if invited, and comments indicated that participants were particularly enthusiastic regarding the feedback component of the interface. As indicated in the previous section, respondents understood the need for using health data to enable medical progress; however, they had previously had very little insight into outcomes of research using health records. The electronic system was in this context considered to be especially valuable in providing a mechanism to enable detailed transparency and feedback on relevant research, which may also improve trust and public engagement in research. For example, the patient quoted in the previous section who said he would opt out viewed the Dynamic Consent interface as a useful tool to improve patient control:

If there’s a trail and you can see where it’s being used...you can find out who is using it, what it’s being used for, and why it’s being used. And then, you know, you could stop it being used.

Participant #15

Participants commented on the positive benefits of gaining feedback of where and when their health data had contributed to published articles or breaking news items (see Textbox 2).

Despite the many positive benefits of the system described and the value of feedback, there were a number of respondents who stated that they would not want to use the system for either consent or feedback. They instead reiterated views that they trusted the NHS and researchers to use their data appropriately:

Well, honestly, [laughingly] I don’t think I would really bother [with the Dynamic Consent interface], but I don’t mind anybody having the information to benefit, you know, other people.

Focus group #1

We’ve got to have research so we can make things better, I mean, what benefit would it be for me to check that feedback. Because they’ve got the information then, and then they know how to treat me...telling me wouldn’t benefit me. Giving me the end product would benefit me.

Participant #28

**Quotations representing transparency through Dynamic Consent and patient feedback, and the potential for enhanced control and patient engagement.**

I like the idea of the Dynamic Consent where you can opt in for bits of it, say, they sent me something online and said we want you to take part in this study, this is what it’ll involve, X, Y and Z...I might say well, I’m happy to do X and Y but not Z...I quite like that.

Participant #3

Well, I think it’s good, you know, to be able to get involved and to be able to track and control what is happening.

Focus group #3

I think a lot more people would like to know where their health information was being used. Some people might refuse getting involved [in research] because there’s a fear of where the information is going.

Focus group #2

[It] lets you know what’s happening. And you might find out it’s [health data] somewhere where you don’t want it to go, but at least you’d know about it.

Participant #12

I just love this idea [Dynamic Consent], the updates they’re great. If I was involved with something [research] and it got published, I could go on the Internet and click on that [dynamic interface] and it would give me all the published papers on it.

Participant #4

I thought, oh, that’s nice to see the actual papers that have been written on things that you contributed to.

Participant #5

I think this [Dynamic Consent] would encourage more people to get involved with research. Yes, definitely it will improve people’s trust.

Focus group #3

### Operational and Technological Scope and Challenges for Participation

Easy usability of the interface was another positive aspect of Dynamic Consent described by participants. In trying out the interface in the focus groups or interviews, many individuals commented that it was easy to use, describing it as "simple and quick" (Participant #16) with the touch screen viewed as "straightforward [for] people with a variety of conditions" (Participant #2). During focus groups and the PPI groups, participants demonstrated that it was easy to use for people with arthritis involving their hands. Some participants, who had no previous experience of using a tablet device, were able to navigate the prototype easily while being directed to various parts of the app. Participants expressed surprise at how easy it was to use, and said they would be enthusiastic to try a live version. However, a number of respondents also expressed a view that they or others, especially older people, may need initial support to be introduced to using the system. A minority of participants (10/40, 25%) described their potential inability to use the Dynamic Consent interface due to either lack of access to IT platforms at home or lack of confidence in utilizing new information technologies. 

These less positive comments were mainly from participants that did not have access to a home computer, never used the Internet, and confessed to being less comfortable utilizing digital technology such as the touch screen interface (see Textbox 3). Out of these 10 participants, 4 (40%) further discussed their willingness to receive support (eg, from a volunteer or a member of staff who could talk through use of the system) to enable use of the system. The remaining few considered they were too old and/or ill—two with terminal cancer—to engage with the technology. A few participants did express they would be happy to complete an alternative paper copy to give consent.

**Quotations representing operational and technological scope and challenges for participation.**

It is, you know, very straightforward, most people could use it, with a variety of conditions, so it’s accessible in terms of that.

Participant #2

Well, it’s very easy to use, isn’t it, the touch screen, it’s a lot easier than a computer.

Focus group #1

It’s hard to take this new technology in, you know, when you get to our age you’re thinking why bother.

Focus group #1

Well, if you show me what to do I’d use it because I’ve never used one, an iPad, you know. As I say, I do use a computer, but not an iPad.

Focus group #1

I wouldn’t know where to start with that [Dynamic Consent interface], I can’t even send a text...I don’t have confidence there...It’s out of my league.

Participant #30

Technology-wise, you know, I think it would be quite interesting if you were into that. I can give you an answer if I was, but, no. For me personally, no, but I'd fill a piece of paper in for you.

Participant #31

## Discussion

### Principal Findings

Patients in this study were highly supportive of sharing their anonymized electronic patient record for research and perceived a Dynamic Consent system for consent and feedback to be valuable if implemented. The three key themes characterized by the participants' views were as follows: trust and social responsibility play a major role in patients’ views about sharing health care data; there is scope for a Dynamic Consent system to facilitate transparency and patient engagement in reuse of health care data that would be highly valued, and would help mitigate concerns about institutional trustworthiness by enhancing individual control and empowerment [26]; and there are some technological and operational challenges for implementing an electronic system for Dynamic Consent. The discussion is structured around these three core themes below.

The findings echo previous research that patients tend to be supportive of the use of their personal health data for research [11,27,28] and reflect a sense of social responsibility and altruism, as well as potential personal benefits associated with medical research [29]. However, while there was a high level of institutional trust in the NHS and health professionals, similar to other studies, there were concerns about security and potential recipients, especially private companies, who might use data inappropriately if it were exported outside of the NHS [14]. Some participants in this study expressed a view that the media were responsible for overinflating the degree of opposition to reuse health data. Nonetheless, views also demonstrated that trust is not universally assumed, and people want reassurance that the conditions underpinning trust are preserved. Such conditions, including values of reciprocity, nonexploitation, and the public good [3], go beyond the established legal framework; current arrangements mean that people lack necessary information and opportunities for greater control over consent and engagement with research based on reused EPRs.

The findings in this study regarding the value of increased transparency and engagement of patients in the reuse of anonymized health care data reflect recent recommendations of the Caldicott 2 review [4] and the recent Nuffield Council on Bioethics report on "The collection, linking, and use of data in biomedical research" [17]. The recommendations aim to "[provide] greater clarity for members of the public about ways that their biomedical data are used" by providing patient-level information about the recipients of their data and the results thereof. Although research is currently conducted using anonymized health care data without consent, few people are aware of this—a finding reinforced throughout our discussions with patients. Such transparency is deemed an important prerequisite for maintaining public trust [16], providing a rationale for greater openness and engagement with patients. The Dynamic Consent system was considered valuable in this respect and could be viewed to enable the black box around consent and the reuse of health care data to be opened.

The initial emphasis when designing the adapted Dynamic Consent prototype for the reuse of health care data, instead of its original purpose of biobanking, was for enabling patients to have greater control over the reuse of their data. However, during the course of the study and analysis of data it has been apparent that patients particularly valued the feedback components enabling greater transparency of how their data were used. They thought this would give insight into previously hidden research of relevance to their health care, which would make them feel valued as participants. There was much less emphasis from participants on the potential for the system to enable greater control regarding consent. This resonates with other research findings that patients valued explicit consent for use of health data and that this was associated mostly with an interest and a curiosity in the kind of research to which they were contributing [14], as well as the opportunity to engage more closely with the research environment [26]. This also aligns with the major emphasis placed on public and patient involvement in research and provision of health care, making patients feel like active participants in a *research active* nation [30]. In practice, provision of feedback on research using EPRs requires an infrastructure that can support an audit trail of which users have accessed the data. It also requires that the system collect lay summaries of the research findings with a link back to the patient participants. Data access agreements would require research groups to upload their results at the end of their studies. No such infrastructure currently exists and would be challenging to implement nationally. However, it aligns well with the recent investment in four national eHealth Centres of Excellence and plans for developing safe havens for health care data for research [2]. Establishing a patient view into such a research infrastructure could deliver a trusting relationship between the patient community and the data repository and its users.

When the touch screen interface was presented, there were examples of enthusiastic engagement, which was balanced against a notable minority who found the technology daunting. This is a common finding in various studies of IT-based interventions to support home monitoring [31] and some have found major barriers associated with nonadoption of IT-based initiatives [32]. Despite some concern about using the system discussed in this study, participants often stated that they would be willing to use the system in a clinical setting if support was provided. Additionally, patients with chronic rheumatic disease can have problems with dexterity and were thus a good population in which to test the touch screen interfaces. While this initial study provided the encouraging results that participants did not demonstrate limitations due to physical functioning, future implementation would need to consider other groups of patients with special needs, such as poor vision. A minority of participants expressed a view that they would not want to use the electronic system even with support because they had no experience or even preferred to avoid using IT devices. However, this is an important issue to consider in planning for implementation of this model of consent and feedback. Because they expressed willingness to engage with aspects of the system—expressing consent preferences and receiving feedback—use of alternative formats or methods for support need further consideration in refining the design of the system. We have since held an implementation workshop with 35 patients to consider further practical issues around implementation. During this positive and supportive meeting, similar and additional considerations were raised, including the need for enabling hands-on support, paper versions of lay research summaries, and tiered options for the depth of information provided to suit varied literacy levels and levels of interest.

The study was conducted among a specific population of patients with chronic rheumatic disease, and thus we need to consider the generalizability of the study. Patients with a chronic disease might be more motivated to share data compared to those with better general health; conversely, a more extensive medical history could make people reluctant to share personal information. Some have reported variations in views regarding requirements for consent that can be influenced by sociodemographic factors and medical history. For example, previous research has shown that patients may be reluctant to share other aspects of health care, such as sexual history or mental health history [33]. Indeed, one participant speculated that people with more sensitive health conditions might be "more guarded of what happens with their health information" (Participant #11). Depression is a common comorbidity in rheumatic diseases such as rheumatoid arthritis [34]. Consequently, our population might represent a group less willing to share their data than the general population. No patients raised concerns specifically about sharing their rheumatology records. Willingness to join the study may have been influenced by an underlying support for data sharing from participants, although it is equally possible that people opposed to data sharing may have been motivated to join. Our experience suggested that few of the participants understood how health care data were currently shared for research and this potential bias is likely to be small. All of our participants were white, reflecting the local demographic. This may bias the study toward more favorable results, as previous studies have suggested privacy concerns may be higher in black and minority ethnic groups [12]. Recruitment from within a clinical setting may have influenced responses toward higher levels of trust. However, views were similar between participants recruited from the clinic and participants from the PPI research network. Implementation of a Dynamic Consent system would need to consider how the setting (eg, touch screens in clinical settings with endorsement from clinical teams versus Web-based systems from home) might influence uptake, engagement, and consent preferences. The implementation plans would also need to extend testing into other population groups.

### Conclusions

In conclusion, this study has generated promising results: a willingness for patients to share their anonymized EPR data for research and a favorable view of a technical solution to meet the needs of recent national recommendations to bring greater transparency and patient engagement in the reuse of EPRs. While uncertainty remains about the degree to which patients will specify consent options in practice, the system offers a potentially valuable technical solution to the challenges of maintaining public trust when sharing medical records for research. This work has provided important insights that will inform the future design of the intervention. We plan to include further codesign [34] in order to maximize the potential for successful implementation and piloting in practice. It represents a first step toward implementation that requires thoughtful development and evaluation, necessarily in a setting with supportive infrastructure. Nonetheless, recent commitment to eHealth research within the government and from funders makes this vision plausible and achievable.

## Abbreviations

EnCoRe: Ensuring Consent and Revocation
EPR: electronic patient record
GP: general practitioner
IT: information technology
MRC: Medical Research Council
NHS: National Health Service
NIHR: National Institute for Health Research
PPI: patient and public involvement
